# Contrast-Enhanced Ultrasonography: Review and Applications

**DOI:** 10.7759/cureus.18243

**Published:** 2021-09-24

**Authors:** Sania Ajmal

**Affiliations:** 1 Department of Radiology, Ascension Genesys Hospital, Grand Blanc, USA

**Keywords:** interventional sonography, microbubbles`, therapeutics in ultrasound, sonography, contrast-enhanced ultrasound

## Abstract

Contrast-enhanced ultrasound (CEUS) has revolutionized ultrasound imaging adding enhanced diagnostic imaging and therapeutic applications to its repertoire. CEUS involves the use of microbubbles which are lauded for their benefit of enhanced imaging without the limitations of radiation exposure or risk of nephrotoxicity seen with other contrast agents. In addition, many innovative uses of microbubbles in diagnosis and treatment stages have been discovered. This article summarizes the composition and resonance properties of microbubbles as the contrast agents for ultrasound, as well as their advanced uses in medicine. The basic role of CEUS is in enhancing the imaging of vessels on a macro and micro level to better classify pathological lesions like atherosclerosis. CEUS is also used in identifying tumor lesions by observing for angiogenesis and monitoring tumors post treatment for remission and relapses. Recent research on using microbubbles for focused drug delivery is very promising. Microbubbles can be used for thrombolysis and targeted delivery of chemotherapeutics in an efficient targeted manner while limiting systemic side effects. CEUS can also be used with therapeutic and diagnostic agents to penetrate into the brain allowing better assessment and management of neurodegenerative disorders. In conclusion, the use of microbubbles opens new frontiers in diagnosis and treatment and will likely be a key technique in medicine in the near future.

## Introduction and background

Contrast agents are regularly used in all forms of imaging such as X-ray, CT and MRI. Ultrasound imaging has also started to benefit from the use of contrast agents that take the form of microbubbles. Microbubbles are bubbles of gas that are smaller than red blood cells. Microbubbles are changing the perception and utilization of ultrasound today. They enable a greater range of diagnostic applications as well as extend the use of ultrasound to therapeutics. With the added benefit of having no nephrotoxicity seen with routine contrasts for CT or MRI. The unique acoustic properties of the microbubbles enable them to produce high-frequency echoes (harmonics) that can be used to enhance the ultrasound images and aid in therapeutic endeavors [1].

The usage of air bubbles as a contrast agent for ultrasound followed a serendipitous observation of Gramiak and Shah [2]. They observed during an echocardiographic examination that agitated saline injection into the left ventricle of the heart produced strong echoes in the aorta. The agitated saline solution had air bubbles that changed the ultrasound image being produced. However, the natural solubility (instability) and size of the gas bubbles limited their use as a contrast agent for the heart [3]. Since then, other types of microbubbles have been found with desirable properties for ultrasonography.

Microbubbles have been improved to increase the time they last in blood. The two major enhancements made to microbubbles are: (i) encapsulating the bubbles in a thin film to prevent the gas or air from dissolving in the blood, and (ii) using gases other than air that take longer to escape the protective film. In 1984 the first stable encapsulated microbubble was produced from human serum albumin insonation. It had the benefit that it could cross the pulmonary capillary network, as its size was comparable to the red blood cells (RBC) [3]. It was commercially marketed as Albunex® (Molecular Biosystems Inc, San Diego, USA). Other agents like Levovist (Bayer, Leverkusen, Germany) were created on a similar principle with a lipid capsule surrounding an air bubble.

Second- and third-generation gas microbubbles were created to increase the stability of the contrast agents in the circulation and enhance their resonance response. These microbubbles (2-6mm in diameter) are dense, hydrophobic gases encapsulated by a shell composed of galactose, albumin, lipid or polymers. A higher density of gases like perfluorocarbons and sulfur hexafluoride makes them last longer in circulation because of their slow diffusion across the capsule membrane and less solubility in the blood, thus increasing microbubble stability over a longer period of time [1,3,4]. The capsule or shell of the microbubbles contributes to its stability and resonant properties.

## Review

Microbubble response to ultrasound waves

Microbubbles resonate when insonated with ultrasound waves, resulting in strong reflection of the acoustic waves because of the high impedance mismatch between the blood, tissues and the bubbles (gas). Microbubble response is highly dependent on the intensity (amplitude) of the incident waves as well as the size and stiffness of the microbubbles. An increase in the amplitude of the incident waves increases the oscillation of the bubbles, whereas an increase in the size and stiffness of the bubble shell suppresses the nonlinear oscillation of the bubbles. The ability of the ultrasound waves to produce microbubble effects is measured using the mechanical index [5]. The mechanical index depends on the peak negative pressure and the frequency of the waves. The peak negative pressure is related to the intensity of the transmitted waves. Different-sized microbubbles may require different frequencies to produce measurable effects. Mechanical index (MI) is calculated based on the following formula:

$Mechanical Index (MI) = \frac{Peak Negative Pressure (MPa)}{\sqrt{Frequency (MHz)}}$

In the United States, Food and Drug Administration (FDA) mandates that MI should be kept below 1.9.

Low amplitude ultrasound beams (mechanical index < 0.07) result in a linear scatter and thus a regular reflection at the same frequency as the transmitted wave. Medium amplitude ultrasound beams (mechanical index 0.07 to 0.3) oscillate the microbubbles faster producing a non-linear response with harmonics. In addition to the incident frequency being reflected, the bubbles also produce multiple high and low-frequency reflections (harmonics, sub-harmonics and ultra-harmonics). Recent ultrasound equipment can detect these harmonic reflections. Harmonic-based imaging improves the bubble to tissue scatter signal ratio, as tissues do not produce significant harmonic backscatter in this range of the mechanical index. This phenomenon makes the bloodstream more opaque compared to the tissues, producing images with greater contrast and thus yielding more diagnostic information. The bubbles because of their size are restricted to the circulatory system and cannot cross the endothelium, which makes them ideal to see vessels and the vascularity of the organs in real-time. Ultrasound waves with higher MI values (> 0.4) burst the bubbles resulting in transient non-linear echoes. It is helpful in bubble detection and tissue perfusion. This difference in bubbles response is also helpful when imaging the perfusion of specific target organs or pathological tissue. It allows us to get a high-frequency image produced by bubble disruption at high MI and then follow it with imaging the tissue to check the rate at which the bubble density is restored [1,3,5]. The characteristics and response of microbubbles to different amplitude beams are summarized in Table 1.

**Table 1:** Microbubble response to ultrasound beams of different amplitude

Amplitude of Incident beam	Mechanical Index	Microbubble response	Acoustic behavior
Low amplitude	< 0.07	Linear oscillation	Linear backscatter
Medium Amplitude	0.07 to 0.3	Nonlinear oscillation	Nonlinear backscatter
High amplitude	> 0.4	Disruption	Transient high energy/non linear echoes

Potential clinical uses of microbubbles

Some promising uses of contrast-enhanced ultrasound (CEUS) are discussed below. The reader is referred to [1,6,7] for example images of CEUS in use.

Identification of Pathological Lesions

Contrast-enhanced ultrasound produces enhanced images of tissue compared to B-mode ultrasound which aids in determining tissue pathology. Its ability to determine tissue perfusion helps determine changes in vascularization in tissue parenchyma secondary to pathological processes or in the pathological lesion in response to treatment. When a tissue is imaged using CEUS it yields basic information about the vascularity of the organ. If the tissue is then imaged under high MI it would result in the microbubble disruption and a high-frequency response [5]. Reimaging the tissue at regular intervals is done to determine the restoration rate of the microbubble density in the tissue vasculature. This helps determine the perfusion rate in the area under observation [6]. It also helps us in determining the effectiveness of cancer treatments targeting the angiogenic properties of tumors [1,6].

Atherosclerotic Plaque Identification and Risk Stratification

The role of contrast-enhanced ultrasound in detecting the degree of atherosclerosis and its use as a tool for risk assessment of coronary or cerebrovascular events has been widely studied in different studies. Atherosclerosis is an inflammation of the vessel wall leading to neovascularization of the vessel intima and plaque formation. Primarily CEUS allows better visualization of the atherosclerotic plaque and the wall of the vessel. Secondly, as the microbubbles are ‘ideal blood pool agents’ they allow us to visualize the degree of neovascularization of the plaques, which has been determined as a marker of plaque fragility and relates to the probability of thromboembolic events in patients [7].

Tissue Specification Helping in Targeted Diagnosis

Tissue specification by microbubbles is currently being done by attaching cell-specific ligands (peptide, carbohydrate or antibody) on the outer shell of the microbubbles [8]. The ligands are targeted to cells receptors that are densely populated on the surface of the diseased endothelial cells. Loading the bubbles densely with multiple receptors for similar cells has shown an increase in the probability of attachment of the microbubbles to the specific tissue. Microbubble contrast agents attached with antibodies to VCAM-1 (vascular cell adhesion molecule-1) or P-Selectin have demonstrated an increased affinity for inflamed endothelium in both advanced and early-stage atherosclerosis in animal studies [7,8]. Similarly, microbubbles with VEGF-2 (vascular endothelial growth factor-2) antibodies show sequestration in sites of vascular proliferation, valuable in detecting cancerous lesions.

Thrombolytic Effect

Contrast-enhanced ultrasound has been found effective in thrombolysis by itself and in conjunction with thrombolytic therapy. When the microbubbles are oscillated at their resonant frequencies, they are disrupted resulting in producing cavitation foci [8,9]. Cavitation results in high-speed microjets which mechanically disrupt the clot and can lead to the full dissolution of the clot [8]. This weakening of the clot also aids the fibrinolytic agents to enter the clot and dissolve it, thus increasing the efficacy of thrombolytic drugs like urokinase and tissue plasminogen activator [9]. Microbubbles specifically targeting the thrombus can lead to an enhanced thrombolytic effect at target sites. Thrombus is primarily formed by platelets, thus peptides with affinity for the 2b/3a receptor of the activated platelets are used to make the bubbles target specific in this case [9]. Advances in the field have led to experiments on thrombus targeting fibrinolytic drug-loaded microbubbles resulting in higher thrombolytic activity with a small dosage of the drug [10]. Clinical application of targeted drug-loaded microbubbles would contribute to better patient outcomes by minimizing ischemic damage to tissues.

Increasing Cell Membrane Permeability

Oscillation of microbubbles by short ultrasound pulses near cell membranes temporarily increases membrane permeability, this process is called sonoporation [8,11]. The following mechanisms explain this phenomenon. Microbubble oscillations produce microjets that can produce small reversible holes in the cell membrane of adjacent cells affecting its permeability [11]. These oscillations also produce intracellular reactive oxygen species and increase the temperature which does not cause cell damage but result in a transient increase in tissue permeability. Alterations in the composition of the lipid bilayer converting hydrophobic pores to hydrophilic temporarily are also thought to contribute to this increase in permeability of the cell membrane. Thus drugs, hormones, genes, etc which cannot diffuse into the cells under normal conditions are able to diffuse into the cells. Clinically, this increases the drug internalization at the cellular level, increasing drug efficiency [11]. Similarly, plasmid DNA carried in microbubbles has been shown to cross cell membranes of the endothelial cells and myocyte by alteration in cell membrane permeability [3,8].

Increasing Blood-Brain Barrier Permeability

Contrast-enhanced ultrasound has proven to be a non-invasive and safe method of altering the permeability of the blood-brain barrier [12]. CEUS however has a dual effect on the blood-brain barrier. When the microbubbles are insonated by focused ultrasound they result in focal regions of increased permeability [8]. This has been found beneficial for targeted delivery of drugs or diagnostic agents to focal lesions in the brain like brain cancers [12].

However, for the detection and treatment of widespread and unknown neurodegenerative diseases of the brain, a more diffuse permeability of the blood-brain barrier is required. Studies have shown that MEUUS (microbubble-enhanced unfocused ultrasound) allows widespread but temporary permeability across the blood-brain barrier. This allows agents (nanoparticles) laden with diagnostic or therapeutic reagents to permeate the blood vasculature and parenchyma of the brain [12]. Nanoparticles are part of the new technology being used for improving brain diagnostics and therapeutics.

Role in Tissue Ablation

In Europe and China, CEUS has been shown to work in conjunction with high-intensity focused ultrasound (HIFU) for tissue ablation [13]. The combination has been shown to increase the efficiency of this non-invasive treatment as well as decrease the time required for the procedure. While the exact mechanism is not well understood it is theorized that the microbubbles cause thermal and mechanical injury at the local level under the influence of high intensity focused ultrasound (HIFU), thus leading to increased tissue destruction in shorter periods of time [13]. Benefits have been seen in benign lesions like fibroids in some trials. CEUS is also useful for monitoring the tissues for incomplete ablation and surveillance for recurrence after treatment by observing the microvascularity in the lesion [6,14].

## Conclusions

Contrast-enhanced ultrasound opens a whole new era for ultrasound in medical applications while being safe and non-invasive. One of the previously acknowledged benefits of CEUS had been the ability to use contrast agents without any nephrotoxicity risk or exposure to ionizing radiations, which had given CEUS an edge over CT and MRI as a tool of investigation and intervention.

One of the key roles of microbubbles is based on their ability to delineate the vasculature including neovascularization, this helps identify atherosclerotic changes in the vessels as well as determining the fragility of the plaques. This property is also being used to identify pathological lesions like tumors due to their angiogenic nature, to monitor the size of such lesions and any subsequent reduction after chemotherapeutic treatment in cancer treatment. Recent interest is in harnessing microbubbles as drug carriers to sites of interest and having localized release by disrupting the microbubbles with high-frequency ultrasound waves. Localized release increases the efficiency of drugs and decreases their systemic side effects. This has been most efficacious for the targeted release of chemotherapeutic agents, circumventing their systemic side effects. A similar benefit has been seen in the thrombolytic effect of microbubbles laden with thrombolytics. Another factor that has enhanced this benefit of microbubbles is their ability to increase the permeability of cell membranes including the blood-brain barrier. Thus, not only the delivery is targeted it allows for more focal penetration of the medications as well. Enhanced blood-brain barrier penetration broadens the horizon for diagnostic and therapeutic benefits to the brain not only for focal lesions but neurodegenerative diseases as well. This article also discusses the role of microbubbles in conjunction with HIFU in tissue ablation by thermal and mechanical injury to lesions.

Although mostly experimental, many clinical trials have shown the efficacy and safety of contrast-enhanced microbubble ultrasound. Once adopted, these advances can change the scope of medicine and the role of interventional ultrasound in it.
